# Perceived behavioral control as a moderator: Scientists’ attitude, norms, and willingness to engage the public

**DOI:** 10.1371/journal.pone.0275643

**Published:** 2022-10-05

**Authors:** Shirley S. Ho, Tong Jee Goh, Agnes S. F. Chuah

**Affiliations:** Wee Kim Wee School of Communication and Information, Nanyang Technological University, Singapore, Singapore; China University of Mining and Technology, CHINA

## Abstract

Scientists play important roles in conducting public engagement, but evidence shows that scientists perceive great challenges in doing so. Drawing broadly from the theory of planned behavior (TPB), this study examines factors predicting scientists’ willingness to conduct public engagement. This study further examines how perceived behavioral control (PBC) of conducting public engagement would moderate the relationships between the proposed predictors and scientists’ willingness to conduct public engagement. Using survey data collected from 706 scientists based in Singapore, this study found that attitude toward and personal norms of conducting public engagement, as well as PBC, significantly predicted scientists’ willingness to conduct public engagement. Notably, PBC interacted with attitude toward conducting public engagement, the perceived descriptive norms, the perceived positive media influence, and the perceived negative external norms of conducting public engagement, as well as personal norms of conducting public engagement to predict scientists’ willingness to conduct public engagement. We postulated the key role that the perception of the ease or difficulty plays in motivating scientists to conduct the skill-intensive endeavor explains the significant moderating effects. The theoretical implications on the TPB and the practical implications for public engagement are further discussed.

## Introduction

Public engagement provides scientists the opportunities to interact with the public on topics that impinge on social progress and public welfare. As a result of rapid changes in science, there is a growing need for public attention and policy interventions in domains such as climate change, energy resources, emerging technologies, food security, and health [1]. Scientists conduct public engagement in domains such as these to defend science from falsehood, share information, nurture interest, and build trust in science (e.g., [2]). In recent years, the intersection of the science-policy interface has propelled the importance of public engagement [3]. However, the practice is not well-defined [4, 5]. Nevertheless, scholars have commonly identified public engagement to constitute two-way communication between scientists and the public through various communication channels to achieve their goals [6–10]. Two-way communication in public engagement emphasizes information exchanges and knowledge co-productions with scientific and non-scientific perspectives [11]. With growing attention on the ethical, legal, and social implications (ELSI) of science, there would be greater expectation for public engagement [12], in which case scientists would assume more important roles in informing the practice of science with input from the public.

Amidst calls for strengthening the rigor of public engagement [13–16], scientists have reportedly faced difficulties in conducting public engagement. Scientists presumed that a lay audience would encounter difficulties in understanding complex concepts [17]. Tailoring an esoteric content for a lay audience could be challenging for scientists who have been accustomed to using scientific jargons [18]. Further, maintaining dynamic exchanges with a live audience requires strong interaction skills [17]. However, scientists’ presumption of their lack of skills, confidence, or experience in using non-scientific terms to discuss their research have reportedly hindered their willingness to conduct public engagement [19–22]. Expansions in the realm of public engagement can also impose higher expectations on scientists. For instance, the heightened societal interest in the ELSI of science calls for adroitness in tackling questions on moral principles. It is conceivable that scientists find themselves unaccustomed to topics beyond their expertise [21]. Scientists have also reflected having insufficient time outside of their teaching and research duties to conduct public engagement; the limited funding and organizational support for public engagement is another common criticism [23]. Since the challenges of public engagement could arise from scientists’ perception of the ease or difficulty of meeting the requirements of effective public engagement, scientists who think that they are adequately equipped in terms of skills and time, and who perceive receiving sufficient support from their research institutes may be more willing to do so [7, 24–28]. Research into the relationship between scientists’ perceived ease or difficulty of conducting public engagement and their willingness to do so has applied the construct of perceived behavioral control (PBC) or self-efficacy [29]. PBC refers to the perception of the ease or difficulty of performing a behavior [30]; it is conceptually similar to self-efficacy [31].

Against the backdrop of the growing significance of public engagement, this study draws broadly from the theory of planned behavior (TPB; [32]) to examine scientists’ willingness to conduct public engagement. Although the TPB has received criticisms for its narrow focus on rational reasoning [e.g., 33], our rationale for drawing broadly from the TPB stems from the understanding that the theory constitutes a known set of constructs (i.e., attitude, social norms, and PBC) that has been applied widely for studying human social behaviors [34]. Meta-analytic studies of the TPB in pro-social organizational contexts have provided evidence of the predictive powers of the constructs [35, 36]. Another meta-analysis of TPB results across different contexts in two countries also showed that there were moderating effects by the PBC in addition to the direct relationships among attitude, perceived subjective norms and behaviors [37]. Together, these findings provide the foundation for this study to test the moderations by PBC.

Executing public engagement effectively constitutes having relatively well-formulated plans to achieve the goals for engagement. Qualitative studies have found that scientists’ attitude toward public engagement, their perceptions of the norms for public engagement within their fraternity, as well as their perceived self-efficacy in conducting public engagement are considerations that can motivate or deter them from doing so [38–41]. Quantitative studies in public engagement have also found support for the TPB through these constructs (e.g., [7, 24, 28]). Based on the meta-analytic evidence of the predictive power of TPB antecedents and the moderating effects of the PBC, we argue that the TPB is an appropriate overarching theoretical framework for the current quantitative study that not only aims to predict Singapore scientists’ willingness to conduct public engagement using similar antecedents, but also intends to examine moderation effects within the established framework.

This study examines scientists’ willingness to conduct public engagement as the criterion variable. Willingness refers to an individual’s openness to an opportunity to perform a behaviour [42]. Although behavioral science research undertaken under the TPB framework has conventionally examined behavioral intention as the criterion variable, this study makes a case for examining willingness. It is arguable that public engagement is usually presented to scientists as *opportunities* that are outside of their core duties than as job requirements since they are more likely to have defined job scopes in research or teaching, or even both [5]. Therefore, it would be more appropriate to apply the construct of willingness to capture the relatively more spontaneous reactions toward public engagement rather than the more rational process of developing behavioral intention [43]. The predictors of the criterion variable are attitude, perceived social norms, personal norms, and PBC. We will delineate the relationships between the predictors and the criterion variable in the literature review.

This study also aims to examine how PBC would moderate the relationships among the abovementioned predictors and willingness to conduct public engagement. This is a novel approach in the public engagement literature that has applied the TPB. Although extant literature has shown how PBC influenced the willingness to conduct public engagement, there is a research gap in testing the moderating effects of PBC in this context. Compared with PBC, which can be raised through programmatic interventions, attitude and norms are relatively more stable individual-level constructs. The results of the moderation would be useful for informing the stakeholders of public engagement whether at the current attitudinal states, and levels of social and personal norms, how low levels of PBC or efforts at raising PBC would affect scientists’ willingness to conduct public engagement. Theoretically, our approach can contribute to the growing literature on the moderating effects of PBC within the TPB framework from a public engagement perspective.

## Context of study: Singapore

Science and technology is a cornerstone of Singapore’s development. In 2010, the government envisioned shaping Singapore into a knowledge-based economy and raising its global competitiveness through research, innovation, and enterprise. Besides manufacturing, the government also identified urban sustainability, health, and digitization as the areas that required deeper research and development (R&D; [44]). The government’s pragmatic approach set science and technology as a pillar for other sectors and a foundation for the later milestones. Developments in public engagement accompanied the further integrations of science and technology into the economy. Scientists in Singapore have conducted live demonstrations at science fairs, presented at science cafes, and interacted with their audiences over traditional and social media [41]. A big stride in formal education was the joint offer of the Master of Science in science communication by the National University of Singapore (NUS) and the Australian National University in 2009. The NUS and the Nanyang Technological University began offering credit-bearing courses on public engagement in the mid-2000s [45]. Science and technology institutes also provided public engagement trainings for scientists (e.g., [46]). The developments of formal public engagement trainings in Singapore attest to the importance of strong institutional support for tertiary education in science communication. Conversely, such programs are lacking in developing countries such as Africa, as the nexus between scientific research and public engagement of science is seemingly lacking [47].

However, a pioneering body of research on public engagement in Singapore presented evidence of scientists perceiving themselves lacking the time, skills, and organizational support for executing what they also believed to be challenging endeavors [41, 48]. One study observed that scientists were less proficient in conducting public engagement than their counterparts in the arts, humanities, and social science disciplines [48]. These observations were made despite scientists largely maintaining a positive attitude toward public engagement. Further, the junior scientists and those who reported having little experience in public engagement reflected having greater difficulty in conducting public engagement than the senior scientists and those with past experiences in public engagement. Likewise, those who had not undergone training in public engagement perceived the task to be more difficult than those who had [37]. Overall, scientists in Singapore feel constrained by perceptions of their own limited abilities and the lack of strong organization support for public engagement.

Scientists in other countries also face the same constraints when conducting public engagement. For instance, between 2014 and 2016, scientists in Italy consistently perceived a lack of communication skills for public engagement [49]. African scientists also faced the same challenges, especially in their abilities to engage in meaningful exchanges with their audiences, raise their enthusiasm toward a science topic, and present with clarity to prevent themselves from getting misconstrued [47]. Situations that require scientists to tailor contents for educational purposes pose further challenges as they could lack pedagogical skills [50]. The reflections by Portugal’s scientists on their weak motivation [51] sum up the implication of the lack of skills on public engagement.

Amidst the leaps that Singapore has made in public engagement, evidence of scientists’ reflections on their inadequacy in conducting public engagement calls for deeper research into the impact of the perception and recommendations for interventions. Therefore, this study positions itself at the point when the public engagement scene in Singapore requires more insights to help keep pace with the expectations for more public engagement. On one hand, the thrusts for R&D to propel Singapore into new stages of development can create opportunities for seeking input for research and policy-making through scientists-public exchanges. The government’s support for knowledge-creation and -sharing through academic research [44] also signals expectations for more public engagement. On the other hand, there appears to be room for improving scientists’ perception of their ability to conduct public engagement, although some scientists perceived themselves to be more efficacious in this regard. On the same note, researchers of the public engagement scene in Singapore have highlighted the need for scientists to bridge the gap between science and the public [52]. The apparent gap in perceived self-efficacy that has appeared amidst the headway that Singapore has made in improving the robustness of the scene for public engagement offers an appropriate context to examine the moderating effects of PBC. This study is also timely in that the empirical findings would carry practical implications for stakeholders who wish to strengthen scientists’ willingness to conduct public engagement.

## Literature review

### The theory of planned behavior

The TPB is a highly-established framework that has explained behavioral changes in the contexts of health, information technology, and environmentalism, among others [48]. Conventionally, the TPB comprises the attitude, subjective norms, perceived behavioral control and behavioral intention constructs [30]. Drawing broadly from the TPB, we posit that attitude, dimensions of perceived social norms, personal norms, and PBC are the key predictors of willingness to conduct public engagement; PBC would also be a viable moderator of the other relationships with the criterion variable. The broad application of the TPB to examine scientists’ willingness to conduct public engagement is consistent with past studies. In this study, we explicate perceived social norms into (a) perceived descriptive norms, (b) perceived positive media influence, and (c) perceived negative external norms of public engagement.

### Attitude toward public engagement

Adding to the definition of PBC provided earlier, we define attitude as the positive and negative evaluations of an entity [53]. Past studies found support for the relationship between attitude and willingness to conduct public engagement. Scientists who held positive attitude toward public engagement in terms of perceiving the act to be socially beneficial [54] and believing that performing it would advance their careers expressed stronger willingness to do so [55]. Among scientists in the United Kingdom, a positive attitude was positively associated with the willingness to conduct public engagement [7]. Similarly, the positive attitude among microbiologist in the United States was predictive of their willingness to conduct face-to-face and online engagement [28]. Drawing on these studies, we hypothesize:

H_1_: Favorable attitude toward public engagement is positively associated with scientists’ willingness to conduct public engagement.

### Perceived norms of public engagement

Perceptions of social norms refers to perceptions of social pressure to act in acceptable manners [56]. One distinctive feature of the studies that applied the TPB to predict scientists’ willingness to conduct public engagement is the addition of relevant antecedents. Scholars have examined whether scientists’ perceptions of social norms that arise from the positive and negative reactions toward public engagement predicted their willingness to conduct public engagement. The common proposition is that a strong perception of what are normative in scientists’ working environments would encourage them to do so. The following sub-sections discuss the relationships among perceived descriptive norms, as well as the norms that arise from the perceived benefits and negative external norms of public engagements, and scientists’ willingness to conduct public engagement.

#### Perceived descriptive norms

Perceived descriptive norms refers to the beliefs about the pervasiveness of certain behaviors [56]. This implies that descriptive norms provide explicit information on the prevalence of common behaviors. Past studies suggested that the perceived descriptive norms of public engagement was inconsistent in predicting scientists’ willingness to conduct public engagement. For instance, the positive associations between perceived descriptive norms and scientists’ prioritization of defending science and providing information through online communication [2] suggested a strong likelihood of conducting online public engagements. On the contrary, one study on scientists who were based in the United States found that the perceived descriptive norms was not associated with the willingness to engage with the lay public online [26]. Interestingly, there was a negative relationship between the perceived descriptive norms and willingness to engage in face-to-face communication—a finding which scholars attributed to the microbiologists’ stronger motivation to fill the void left by their co-workers [28]. To probe further into the influence of perceived descriptive norms on scientists’ willingness to conduct public engagements, we pose the following research question:

RQ1: How does the perceived descriptive norms of public engagement influence scientists’ willingness to conduct public engagement?

#### Perceived positive media influence

Scientists’ perceptions of positive media influence on their careers can set up norms for conducting public engagements. The presumptions of positive media influence, in terms of gaining recognition and resources, and facilitating their promotion, can motivate scientists to be more active in conducting public engagements. Recognizing the potential boosts to their careers, scientists could expect themselves to be more devoted to public engagement. Expectations are related to norms in that normative actions in a group signal beliefs on the standard that members should meet [56]. Hence, with strong perceptions of positive media influence on their careers and research, there could be strong expectations within scientific research communities to meet the standards of conducting public engagement. This could establish strong norms for partaking in public engagement.

Scientists presumed positive media influence on their careers in many ways. Studies found that scientists were motivated by the possibility of enhancing reputation [19] and raising the prominence of their research centers [57] through public appearances. Another study found that scientists who conducted research in nanotechnology were motivated by the prospects of academic citations as indicators of others’ recognition of their work [58]. The possibility of subsequently attracting research funding and talents through heightened exposure added to the presumptions of positive media influence. Japanese scientists’ acknowledgement that there were personal and career benefits ([59]; p. 9) to carrying out public engagement reinforces the point on scientists deriving some personal benefits, such as greater confidence of their own abilities, from their career successes. As these findings indicate that scientists recognized the positive media influence, the norms to conduct public engagement may be strong. Therefore, we posit:

H_2_: Perceived positive media influence is positively associated with scientists’ willingness to conduct public engagement.

#### Perceived negative external norms

On the other hand, research has shown that perception of negative external norms can shape scientists’ perception that public engagement is socially disapproved. Receiving sanctions for not adhering to social standards set up perceptions of social norms [56]. Sanctioning public engagement within the research fraternity can give scientists the impressions that there are norms—both explicit and implicit—about scientists’ fulfillment of their responsibilities as researchers vis-à-vis public communicators. In other words, it connotes that public engagement is secondary to research. When scientists conduct public engagement to the extent of failing to adhere to the norms of fulfilling their main research duties, they may face disapprovals from their organizations. Studies found that scientists perceived such negative external norms against public engagement: prioritizations of research output over public engagement and relatedly the lack of recognizing public engagement efforts in work evaluations [41]. Colleagues’ disapprovals of public engagements [7] also give scientists the impressions that their efforts would be viewed negatively. These revelations were indicative of the sanctions that contributed to perceptions of unfavorable organizational norms toward public engagements. Guided by these findings, we posit:

H_3_: Perceived negative external norms is negatively associated with scientists’ willingness to conduct public engagement.

### Personal norms of public engagement

Past studies have also examined how personal norms influenced the willingness to conduct public engagement. This initiative is in line with scholarly recommendation that examining personal norms is a useful extension of the TPB [60]. Personal norms refers to one’s moral values. It imposes sanctions on the self for non-compliance with one’s beliefs [61]. Anticipation of guilt for failing to adhere to one’s beliefs can drive individuals to act in accordance with their personal norms [62]. In this sense, personal values can set expectations for the self to perform a behavior.

Research showed that scientists’ personal norms for public engagement manifest in various ways. Personal values such as altruism [38], a sense of moral obligation to share important information [63], and a desire to repay taxpayers by sharing the results of publicly-funded research [64] were reflective of scientists’ strong personal norms for public engagement. Scientists who opined that public engagement can contribute to social welfare held better attitude toward the behavior, and therefore reported higher levels of past engagement [25]. The more scientists enjoyed sharing the value of their research, the more frequently they engaged with journalists [27]. Scientists’ sense of public duty to conduct public engagement over the media also motivated them to do so [9]. Based on the strong evidence related to personal norms, we posit:

H_4_: Personal norms is positively associated with scientists’ willingness to conduct public engagement.

#### Perceived behavioral control

Individuals with stronger PBC are presumably more successful at enacting a behavior because they feel more confident about their ability to access the resources required for completing various tasks [65]. Building on the earlier discussion on the areas in public engagement which scientists have faced challenges in, extant literature presents strong evidence that scientists’ perception of self-efficacy for conducting public engagement is predictive of their willingness to conduct public engagement in both online and offline settings [7, 10, 24, 26–28]. Hence, we posit:

H_5_: PBC is positively associated with scientists’ willingness to conduct public engagement.

#### The moderating effects of perceived behavioral control

The literature presents two main ways of interpreting the TPB. First, the traditional argument is that attitude, subjective norms, and PBC have an *additive* function on behavioral intention [34]: if any antecedent is weak, it is still possible to form behavioral intention as long as the other two antecedents can compensate. For instance, individuals who display an unfavorable attitude toward a behavior can still develop strong behavioral intention as long as the subjective norms and PBC are sufficiently high. The second argument is that the PBC can serve as a moderator, which is one of the initial propositions in the formulation of the TPB [66]. Beyond a simple additive effect of the antecedents, scholars have argued that PBC can influence the strength of the attitude-intention and subjective norms-intention relationships [53, 67]. Adopting the latter approach can add new perspectives to the literature on public engagement since scientists’ willingness to conduct public engagement could be related to their perceived ease or difficulty of the behavior.

Scholarly discussions on PBC have shaped knowledge on its role as a moderator in the TPB. The anecdotal account of how an intention to quit smoking would be low as long as smokers believe that they lack the ability to do so, even if they bear positive attitude about the health outcomes and are highly aware of others’ expectations to do so, highlights the moderating potential of volition [68]. Further, Fishbein and Ajzen reiterated the proposition that “PBC is expected to moderate the effects of attitudes and social norms on intentions…” ([69]; p. 181). However, due to the lack of empirical support, the moderation proposition failed to receive as much attention as the additive proposition [70]. In other words, the small volume of research that examined the moderating role of PBC stems from the lack of empirical support, not the lack of theoretical support. The paucity of studies that investigated the moderation effects of PBC implies that scholars have missed opportunities to understand the motivators of behavioral intention at a deeper level. These perspectives on PBC provide an impetus for this study to examine its moderating potential since scientists’ public engagement is likely an endeavor that is dependent on perception of being sufficiently skilled.

Recent studies that examined the moderating role of PBC found preliminary support for this notion. Studies found that in the case of intention to quit smoking, attitude and subjective norm were more strongly related to intention when PBC was high [68]. PBC also interacted with the association of attitude and personal norms with six cancer-screen and health-enhancing intentions [71]. The interaction effects were also observed in undesirable behaviors. For example, PBC moderated the relationship between attitude and intention to consume cannabis [72, 73], intention to tailgate, and intention to violate traffic rules [74]. Overall, there is some evidence to suggest that PBC moderates the relationships between attitude and intention, and norms and intention.

In line with the aim of our study, we extend the existing research on the moderating effects of PBC on behavioral intention to examines whether PBC will moderate the relationships between the antecedents of scientists’ willingness public engagement and the variable itself. Therefore, we pose the following questions:

RQ2: Does PBC moderate the relationship between positive attitude and scientists’ willingness to conduct public engagement?
RQ3: Does PBC moderate the relationship between perceived descriptive norms and scientists’ willingness to conduct public engagement?
RQ4: Does PBC moderate the relationship between perceived positive media influence and scientists’ willingness to conduct public engagement?
RQ5: Does PBC moderate the relationship between perceived negative external norms and scientists’ willingness to conduct public engagement?
RQ6: Does PBC moderate the relationship between personal norms and scientists’ willingness to conduct public engagement?

## Method

Data collection took place between July and August 2018 through an online survey. After obtaining ethical approval, we sent 2,662 invitation e-mails to the scientists working in Singapore’s public universities and public research institutions. We sent the e-mails in four batches. After sending the initial e-mail, we followed up with two reminders in two-day intervals. Respondents could access the survey by clicking on the link stated in the e-mails. We offered S$20 worth of shopping vouchers to the respondents who completed the questionnaire as tokens of appreciation.

### Ethics statement

The study protocol was approved by the Institution Review Board (IRB) of the Nanyang Technological University (IRB number: 2017-03-009-02). All participants provided their written consent to take part in the study by answering an informed consent form.

### Sampling

#### Sampling procedure

To create the sampling frame, we compiled an exhaustive list of researchers in the Science, Technology, Engineering, and Mathematics (STEM) fields in Singapore by going through the websites of the six public universities and 19 public research institutions. We randomized the list before sending the invitation e-mails. To be eligible for the survey, the respondents had to meet three criteria: (a) hold a Ph.D. degree, (b) employed by a public university or public research institution in Singapore, and (c) conduct research in STEM fields. To ensure that the respondents meet these criteria, we required them to answer screener questions at the start of the survey.

#### Sample

With 706 completed surveys, we attained a 40.9 percent response rate (based on formula 3 for response rate by the American Association for Public Opinion Research). Specifically, 336 of the respondents were employees of public universities and 370 were employees of public research institutes. In terms of seniority, 47.7 percent of them held senior positions (i.e., Professor, Associate Professor, Senior Research Fellow, Senior Principal Investigator, Senior Scientist, and Senior Research Engineer), while 52.3 percent held junior positions (i.e., Assistant Professor, Research Fellow, Junior Principal Investigator, Junior Scientist, Junior Research Engineer, and Research Associate). The respondents had between 0.3 and 45 years of research experience in Singapore (*M* = 10.2, *SD* = 8.05). They represented 35 disciplines, which we later categorized into science, technology, engineering, and others. In terms of gender, 75.4 percent of them were male and 24.6 percent were female. The age range was 26 to 73 years old (*M* = 40.9, *SD* = 9.45). When it comes to nationality, 317 of them were Singaporeans, 164 were permanent residents, and 221 were foreigners. Four respondents did not indicate their nationality. The distribution of the sample enabled us to examine the public engagement of scientists of various disciplines and levels of seniority.

### Measures

We included nationalities, gender, countries from which PhD degrees were attained, seniority, and tenure-track positions as control variables. We created dummy variables for the countries where more than 50 participants attained their PhD. Four countries fit this criterion: China, Singapore, the United Kingdom, and the United States. Another dummy variable was created for participants who attained their PhD from countries aside from these four main countries. S1 Table shows the complete wordings and descriptive statistics of all the variables. S2 Table provides the zero-order correlations of all variables. Unless stated otherwise, we measured all variables using a 5-point Likert scale and averaged the items to create composite indices. A higher score indicates a higher value of each of the following composite measures.

#### Willingness to conduct public engagement

Participants indicated, through 16 questions, their willingness to conduct various types of public engagement activities (e.g., “In the next 12 months, I am willing to be interviewed by media targeted at the non-expert public on science topics,” and “In the next 12 months, I am willing to provide science knowledge to the public relations department of research institutions.”). The questions were adapted from past studies [25, 75]. A composite measure was constructed (*M* = 3.72, *SD* = 0.72, Cronbach’s α = .92).

#### Attitude toward public engagement

Participants indicated their attitude toward communicating their research findings through four questions that were measured on a 5-point semantic differential scale (e.g., “Communicating my research findings to the non-expert public is bad: good.” and “. . .unenjoyable: enjoyable.”). The questions were adapted from a past study [7]. A composite measure was constructed (*M* = 4.10, *SD* = 0.77, Cronbach’s α = .90).

#### Perceived descriptive norms

Respondents indicated how much they agreed or disagreed with three statements on the perception of the norm of taking part in public engagement among their colleagues, superiors, and other scientists in Singapore (e.g., “My colleagues/superiors take part in public engagement of science.” The questions were adapted from a past study [58]. A composite measure was constructed **(***M* = 3.63, *SD* = 0.83, Cronbach’s α = .84).

#### Perceived positive media influence

Respondents indicated how much they agreed or disagreed with five statements on the perceived positive influence of taking part in public engagement (e.g., “Universities find it more difficult to deny tenure to candidates who make frequent media appearances.” and “Media appearances help researchers get funding.”). The questions were adapted from the same study mentioned above. A composite measure was constructed (*M* = 3.55, *SD* = 0.70, Cronbach’s α = .79).

#### Perceived negative external norms

Respondents rated the importance of three considerations for the performance of public engagement (e.g., “Possible critical reactions from other scientists in the same field/my supervisors.”). The questions were adapted from the same study mentioned above. A composite measure was constructed (*M* = 3.77, *SD* = 0.85, Cronbach’s α = .74).

### Personal norms

Respondents indicated how much they agreed or disagreed with four statements on the personal norms to perform public engagement (e.g., “I feel a personal obligation to engage in public engagement of science.” and “I feel guilty when I turn down public engagement requests.”). The questions were adapted from past studies [7, 76]. A composite measure was constructed (*M* = 3.52, *SD* = 0.87, Cronbach’s α = .84).

#### Perceived behavioral control

Respondents indicated, through four questions, how confident they were when carrying out public engagement (e.g., “Explain scientific facts in a way the non-expert public can understand” and “Adapt to different groups of people who are non-expert”). The questions were adapted from past studies [7, 58]. A composite measure was constructed (*M* = 3.90, *SD* = 0.71, Cronbach’s α = .88).

### Analytical approach

We analyzed the data using ordinary least squares (OLS) hierarchical regression in *SPSS Version 25*. Prior to performing the regression analysis, we dummy-coded participants’ nationalities, genders, countries from which they attained their PhD degrees, seniority, and whether they were holding tenure-track positions. We entered all the independent variables into the regression model based on their assumed causal order: demographics variables (i.e., the control variables) into the first block, the TPB variables into the second block, and the five interaction terms into the third block. The interaction terms were created by multiplying the standardized values of the independent variables. These interaction terms include the interaction between PBC and (a) attitude, (b) perceived descriptive norms, (c) perceived positive media influence, (d) perceived negative external norms, and (e) personal norms.

## Results

We conducted OLS hierarchical regression to predict scientists’ public engagement. Table 1 presents the zero-order correlations and models used for predicting the outcome.

**Table 1:** Factors predicting scientists’ willingness to conduct public engagement.

	Model 1	Model 2	Model 3
**Block 1: Control variables**			
Nationality (1 = Singaporeans; 0 = Others)	.01	.04	.03
Gender (1 = Male; 2 = Female)	-.04	.01	-.01
Attained PhD from America (1 = Yes, 0 = No)	.02	.03	.03
Attained PhD from United Kingdom (1 = Yes, 0 = No)	-.01	-.01	-.01
Attained PhD from China (1 = Yes, 0 = No)	-.10*	-.14***	-.14***
Attained PhD from other countries (1 = Yes, 0 = No)	-.03	-.04	-.04
Seniority (1 = Senior, 0 = Junior)	-.06	-.04	-.04
Tenure (1 = Yes, 0 = No)	.02	.02	.03
Age	-.04	-.11*	-.12*
Experience	-.11	-.06	-.05
Incremental *R*^2^ (%)	3.20*		
**Block 2: Theory of Planned Behaviors**			
Attitude		.36***	.34***
Perceived descriptive norms		-.01	.00
Perceived positive media influence		.01	.01
Perceived negative external norms		.04	.04
Personal norms		.29***	.29***
Perceived behavioral control (PBC)		.21***	.19***
Incremental *R*^2^ (%)		50.40***	
**Block 3: Interactions**			
Attitude*PBC			-.10**
Perceived descriptive norms *PBC			-.08**
Perceived positive media influence *PBC			-.06*
Perceived negative external norms *PBC			-.08**
Personal norms*PBC			-.06*
Incremental *R*^2^ (%)			1.50***
Total *R*^2^ (%)			55.10***

Note: ***p < 0.001

**p< 0.01

*p < 0.05.

The results showed that across all the control variables, attaining a PhD from China was negatively associated with scientists’ public engagement (β = -.14, *p* < .001). In other words, these scientists displayed a lower level of willingness to conduct public engagement as compared to the scientists who received their PhD from other countries. Age was also negatively associated with scientists’ public engagement (β = -.12, *p* < .05), indicating that the younger scientists are more willing as compared to the older scientists. Other demographic variables, such as nationality, gender, seniority, being on tenure track, and years of experience as a researcher in Singapore were not significantly associated with scientists’ public engagement. This block accounted for 3.20 percent of variance in scientists’ public engagement.

As for the TPB variables in block 2, the results showed that attitude toward public engagement was positively associated with scientists’ public engagement (β = .34, *p* < .001). H_1_ was supported. However, perceived descriptive norms, perceived positive media influence, and perceived negative external norms were not significantly associated with scientists’ public engagement. In response to RQ1, perceived descriptive norms was not predictive of scientists’ public engagement. H_2_ and H_3_ were also not supported. The results also showed that personal norms (β = .29, *p* < .001) and PBC (β = .19, *p* < .001) were positively associated with scientists’ public engagement. Therefore, H_4_ and H_5_ were supported. This block explained 50.40 percent of the variance in scientists’ public engagement.

**Fig 1:**
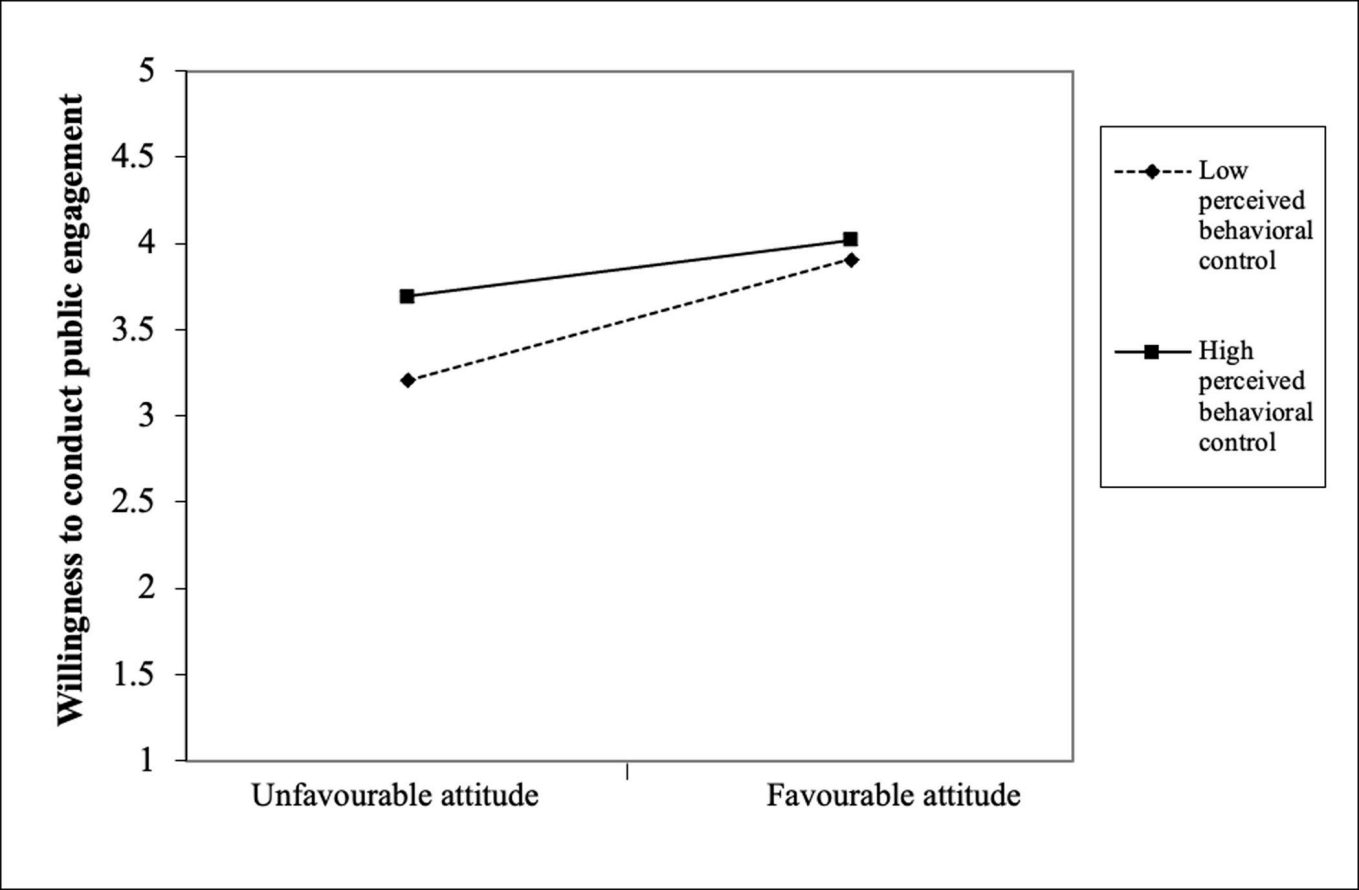
Perceived behavioral control as a moderator of the attitude-willingness relationship.

**Fig 2:**
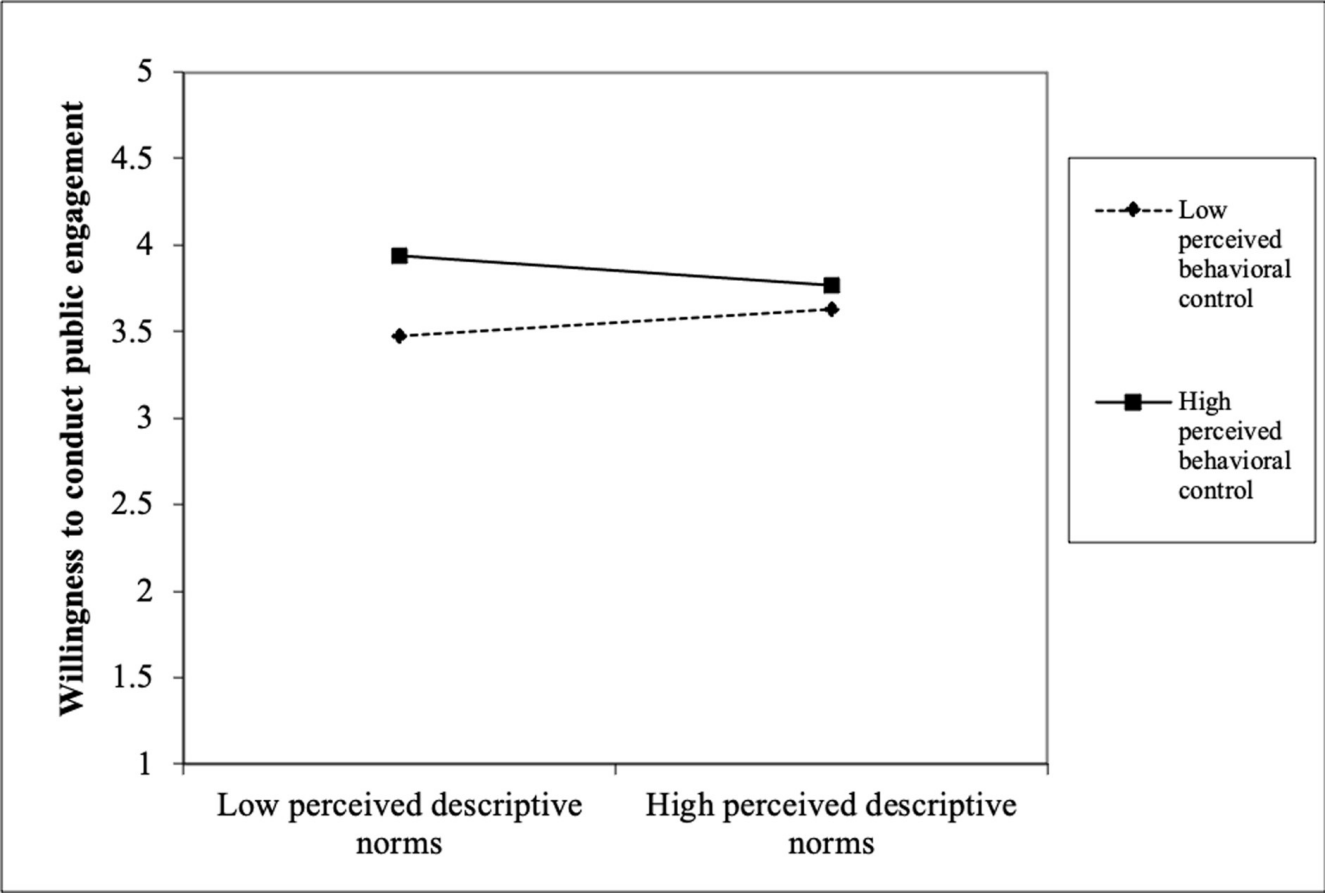
Perceived behavioral control as a moderator of the perceived descriptive norms-willingness relationship.

**Fig 3:**
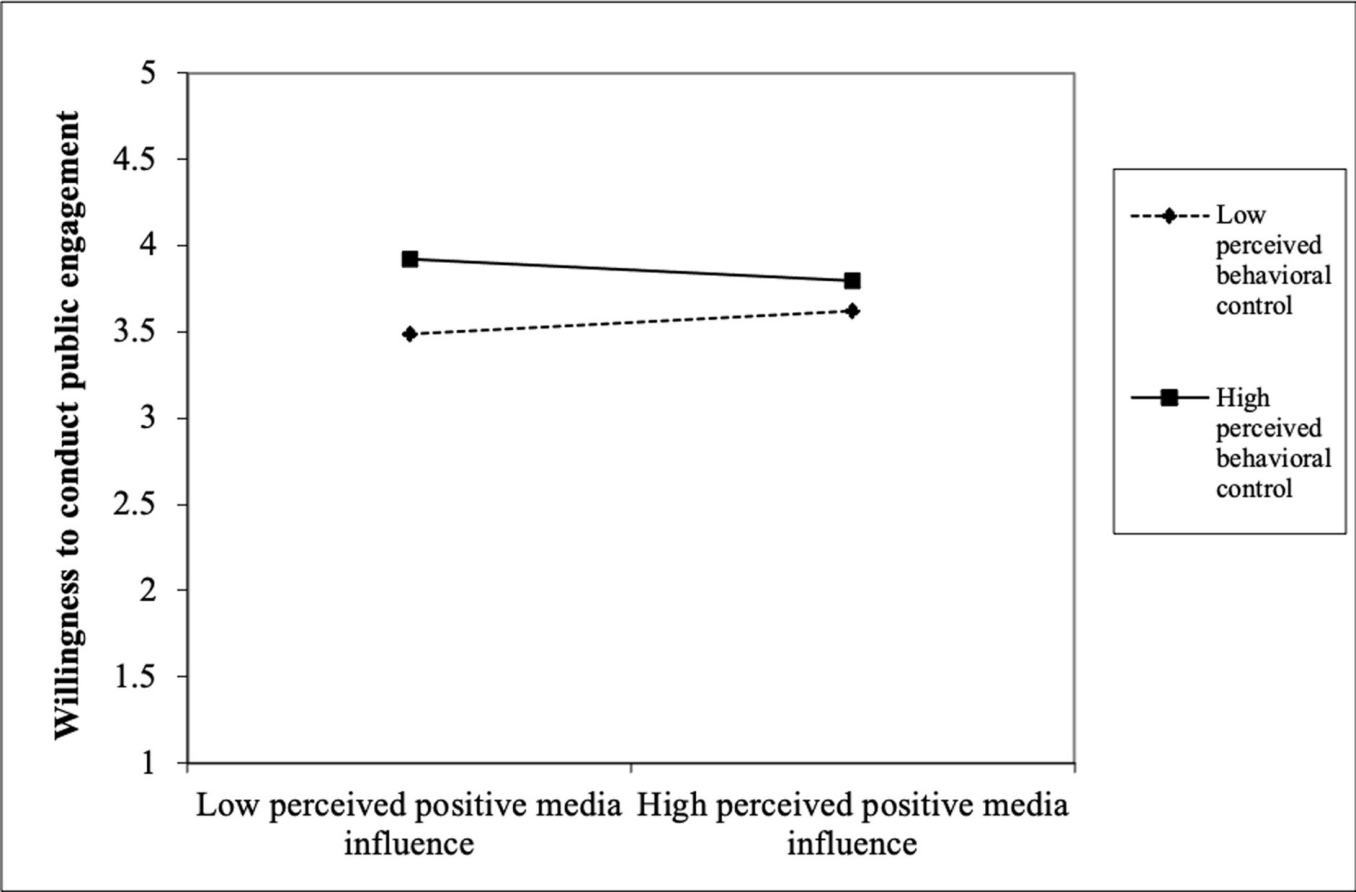
Perceived behavioral control as a moderator of the perceived positive media influence-willingness relationship.

**Fig 4:**
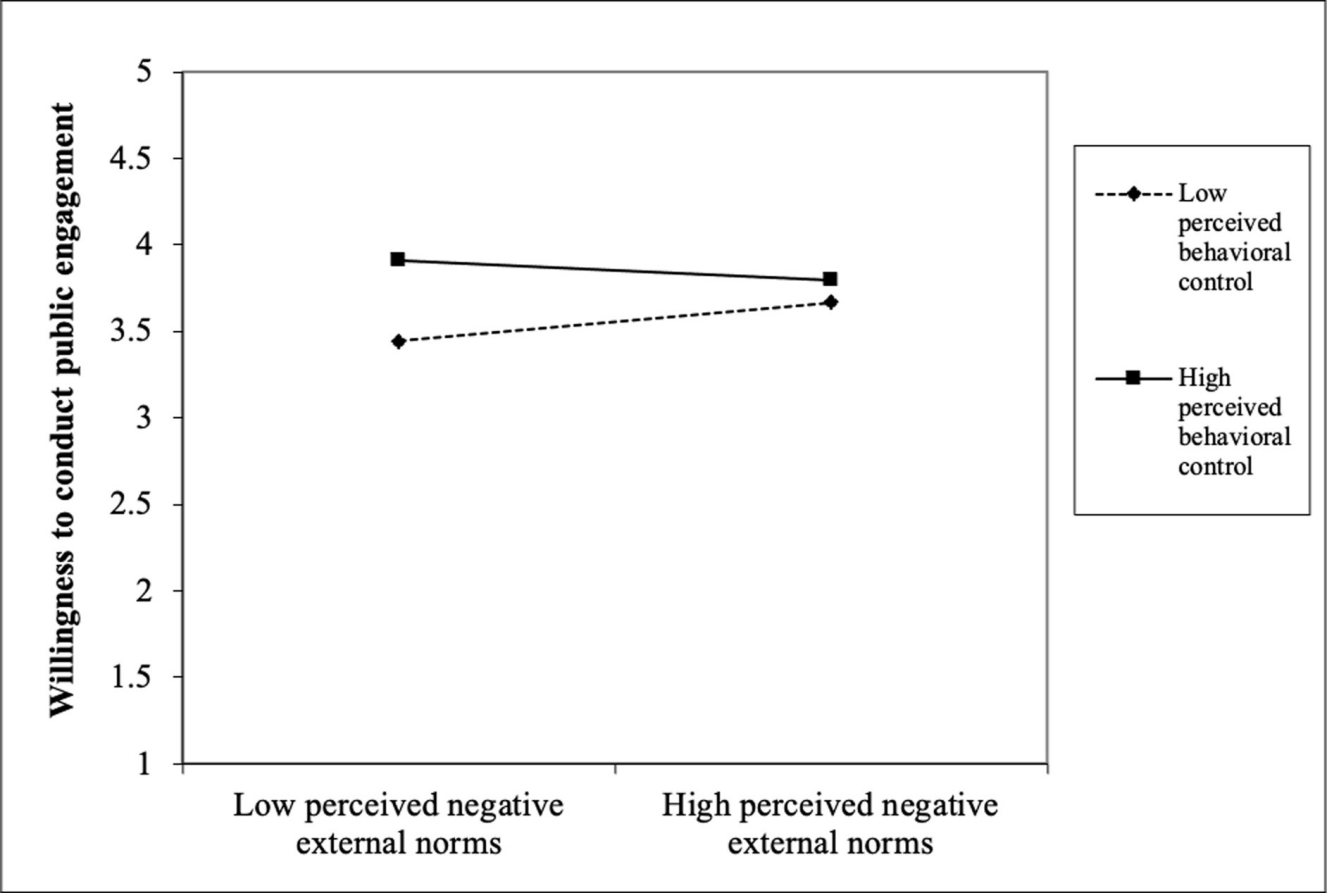
Perceived behavioral control as a moderator of the perceived negative external norms-willingness relationship.

**Fig 5:**
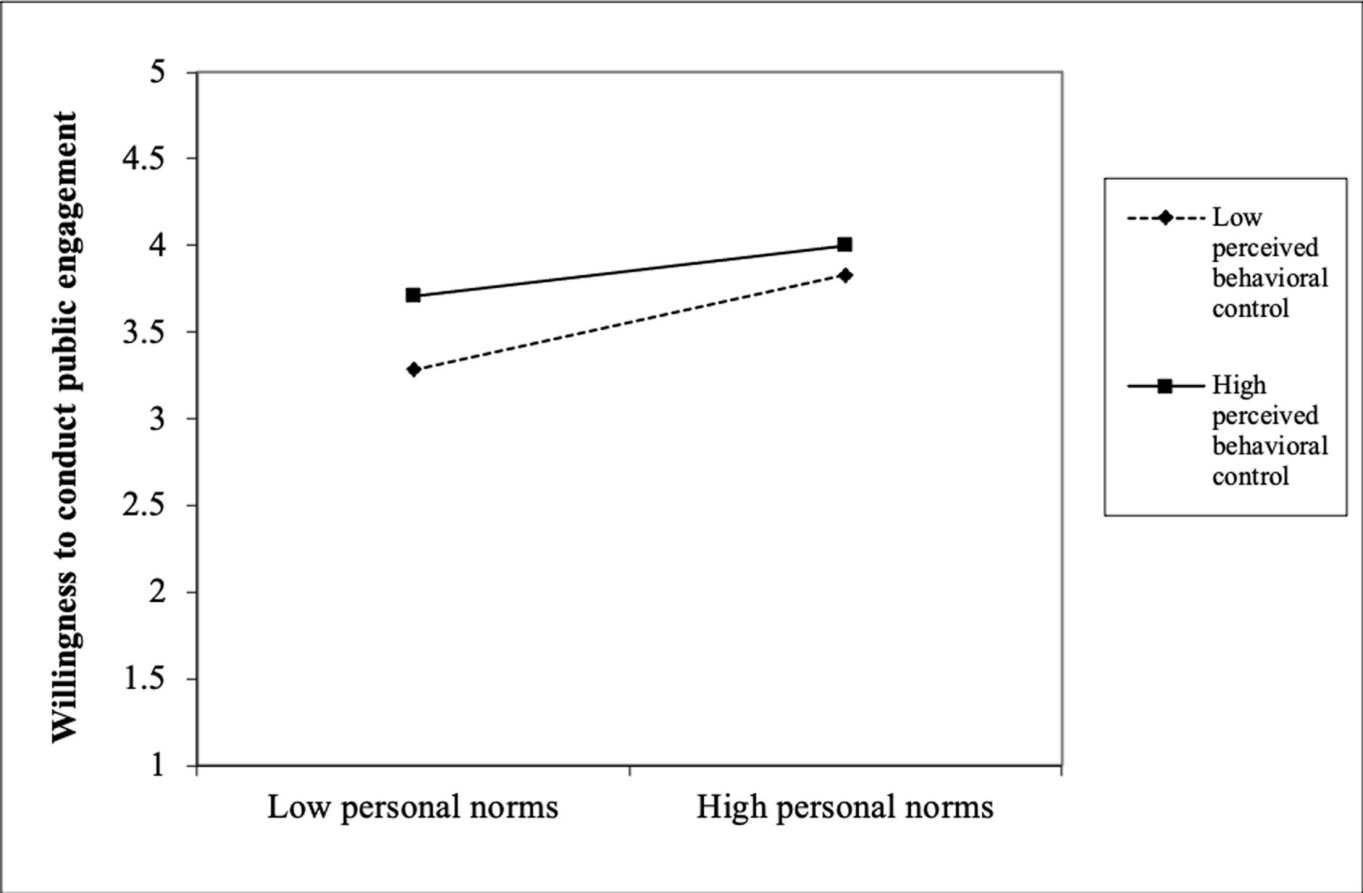
Perceived behavioral control as a moderator of the personal norms-willingness relationship.

Finally, the interaction results showed that PBC significantly moderated the relationships that we tested earlier in block 2. With regard to RQ2, the results show that PBC interacted significantly with attitude (β = -.10, *p* < .01) to influence scientists’ public engagement. Fig 1 shows that among the scientists with unfavourable attitude, those with high PBC were more willing than those with low PBC. However, this difference was not observed among the scientists with favourable attitude. With regard to RQ3, PBC significantly interacted with perceived descriptive norms (β = -.08, *p* < .01) to influence scientists’ public engagement. Fig 2 shows that among the scientists with low perceived descriptive norms, those with high PBC were more willing than those with low PBC. However, this difference was comparatively smaller among the scientists with high perceived descriptive norms. With regard to RQ4, PBC significantly interacted with perceived positive media influence (β = -.06, *p* < .05) to influence scientists’ public engagement. Fig 3 shows that among the scientists with low perceived positive media influence, those with high PBC were more willing than those with low PBC. However, this difference was comparatively smaller among the scientists with high perceived positive media influence. With regard to RQ5, PBC significantly interacted with perceived negative external norms to influence scientists’ public engagement (β = -. 08, *p* < .01). Fig 4 shows that among the scientists with low perceived negative external norms, those with high PBC were more willing than those with low PBC. This difference was not observed among the scientists with high perceived negative external norms. Finally, with regard to RQ6, PBC significantly interacted with personal norms (β = -.06, *p* < .05) to influence scientists’ public engagement. Fig 5 shows that among the scientists with low personal norms, those with high PBC were more willing than those with low PBC. However, among scientists with strong personal norms, the willingness among those with low PBC and those with high PBC did not differ much. This block explained 1.50 percent of the variance in scientists’ public engagement. Overall, the final regression model explained 55.10 percent of variance in scientists’ public engagement.

Table 1 presents the results of the OLS hierarchical regression analysis.

## Discussion

In examining how PBC would moderate the relationships among attitude, norms, and scientists’ public engagement, this study made several key findings. First, this study found that PBC significantly moderated the associations of attitude, perceived social norms, and personal norms with scientists’ willingness to conduct public engagement. Second, we found that personal norms significantly predicted scientists’ willingness to conduct public engagement. Third, the relationships between perceived social norms (i.e., perceived descriptive norms, perceived positive media influence, and perceived negative external norms) and scientists’ willingness to conduct public engagement were non-significant, which are contrary to our hypotheses. These noteworthy results can deepen the theoretical understanding of how PBC would influence willingness to conduct public engagement and how different dimensions of norms in the TPB predict scientists’ willingness to do so. Meanwhile, the results reflected the possibility of strengthening scientists’ PBC as a means to improve public engagement. The following sub-sections discuss the findings as well as the practical and theoretical implications.

### PBC as a significant predictor and moderator

Consistent with past research that we have reviewed earlier, PBC was a significant predictor of scientists’ willingness to conduct public engagement. PBC also significantly moderated the associations of attitude, perceived social norms (i.e., perceived descriptive norms, perceived positive media influence, and perceived negative media influence), and personal norms with scientists’ willingness to conduct public engagement. Unlike the findings of a recent meta-analysis, which found that PBC did not moderate the attitude-intention and norm-intention relationships [77], this study found that among those who scored low on the other three predictors, those who possessed a high level of PBC displayed stronger willingness to conduct public engagement than those with a low level of PBC. A possible reason for the moderation effects among scientists with an unfavorable attitude toward public engagement and a low level of perceived positive media influence is that the perception of ease of conducting public engagement overrode their assessments of public engagement. Conversely, a positive attitude and a high level of perceived positive media influence could have been strong drivers of willingness to conduct public engagement in themselves, to the extent that PBC did not play as strong a role in elevating the willingness. Similar arguments can be made for the moderation of personal norms by PBC: PBC served as a stronger elevator of willingness among those with weak personal norms of conducting public engagement than those with strong personal norms possibly because PBC gave the former group an impression that public engagement was manageable. However, PBC was a weaker driver of willingness in the presence of strong personal norms. PBC could have also elevated the willingness of scientists who rated low on perceived descriptive norms because the perception of having the skills to conduct public engagement could have motivated them to express stronger willingness to conduct more public engagement to make up for an act that they believe to be uncommon within their fraternity. The weaker moderation among those with high perceived descriptive norms could be due to scientists not believing as strongly about the need to do more of what they think is already common or sufficient. The perceived ease of carrying out public engagement could have raised the willingness of scientists who perceived a low level of negative external norms because they were motivated to perform what they believed to be relatively easy tasks that would meanwhile not invite much critical reactions. However, it is plausible that PBC was not as strong a moderator among scientists who perceived a high level of negative external norms as they might perceive that public engagement made scientists susceptible to criticisms.

The finding that PBC strengthened scientists’ willingness to conduct public engagement under the abovementioned conditions presented by scientists is congruent with past findings that PBC was directly associated with scientists’ willingness to conduct public engagement. In the context of this study, PBC manifested in the form of possessing skills for conducting public engagement. Scientists, with their prevailing attitude, as well as perceived social norms and personal norms for conducting public engagement, could have perceived that they would be able to do a reasonable job if they were equipped with the requisite skills. This signals a possibility that the scientists perceived public engagement to be a skill-intensive endeavor, to the extent that the perception of being adequately-skilled could raise their willingness to carry out public engagement. Hence, when interpreted in terms of skills, the results indicated that PBC is a plausible elevator of scientists’ willingness to conduct public engagement; this is especially so among the scientists who were rated lower on the other predictors. In short, PBC can be considered a key enabler for scientists, especially among those who lacked positive evaluations and perceived weak norms of public engagement.

### The norms for public engagement

With the considerations of personal norms and the perceived social norms of public engagement, this study found that scientists’ personal norms significantly predicted their willingness to conduct public engagement. This is a noteworthy finding in relation to the non-significant relationships between scientists’ perceived social norms of public engagement (i.e., perceived descriptive norms, perceived positive media influence, and perceived negative external norms) and their willingness to do so. A possible explanation of this difference is that the scientists could have recognized themselves more as civic scientists who can serve the society’s needs for advancement. In the words of Dr. Neal Lane, the former director of the U.S. National Science Foundation, a civic scientist is someone who utilizes his or her scientific acumen to connect science with the society [78]. Serving this role, scientists can play the leadership or advisory roles in professional science societies, as well as in the educational and political realms (e.g., [79]). Their recognition that the value of science lies not only in its ability to advance the frontiers of science through research and formal education, but also as a service to humanity, could have elicited strong moral obligations for them to realize this potential for the society. With a heightened sense of servicehood, scientists expressed stronger willingness to benefit others with the results of their research through public engagements.

Earlier, we explained that explicit information facilitated the development of descriptive norms. However, the results showed that even perceptions of the explicitness of public engagement could not influence scientists’ public engagement. To explain this outcome, we draw upon the earlier point that scientists could have been more motivated by servicehood to conduct public engagement. We argue for the possibility that scientists felt less convicted by the presumed prevalence of public engagement than by the benefits they can bring to the audience. This is indicative that the value of public engagement for scientists lies less in its popularity, or strong acceptance, among the research community than by the social good that science and public engagement can deliver to society. Likewise, the normative standards of public engagement that arise from perceptions of positive media influence on their careers and from the perceptions of others’ disapproval did not impose as strong a pressure to conduct public engagement as from the conviction to do so based on servicehood. In other words, the perceptions of personal gains and losses of conducting public engagement were not compelling motivations for doing so. Taken together, these findings reinforced the possibility that scientists perceive public engagement more of a moral duty than tasks to be fulfilled in their list of work duties.

The results also draw focus on the distinction between the intrinsic and extrinsic motivations for public engagement. From the perspective of the self-determination theory [80], the positive association between scientists’ personal norms for conducting public engagement and the willingness to do so can be interpreted as the intrinsic motivation for conducting public engagement. In this sense, the scientists could have been driven by their understanding of the worth and purpose of civic science to conduct public engagement. This could be related to their attitude toward public engagement (i.e., their evaluations). However, the perceived tangible rewards and consequences of public engagement—delineated through the constructs of perceived negative media influence and perceived positive media influence in this study—appeared to have no direct effects on scientists’ motivation. While we cannot make extensive statements about the relevance of extrinsic motivations of public engagement through this study, one important takeaway is that scientists could have been introspective of the importance of civic science and motivated by the enjoyment of public engagement for its intrinsic value. Drawing the relation to the prevailing discussion on the objectives that scientists set for public engagement [2, 81, 82], our proposition on civic scientists—made through our findings on the norms for public engagement—opens the discussion to further inquiries on how the intrinsic motivations of aspiring civic scientists or those who identify themselves as one shape their goals for public engagement for various disciplines of science.

### Theoretical and practical implications

The significant direct effects and moderating effects of the PBC contribute to the theoretical understanding in the context of TPB. First, this study lends support to the additive function of PBC in the TPB. Also consistent with scholars’ propositions on the ability of PBC to moderate other relationships in the TPB, PBC moderated the attitude- and norms-willingness relationships in the context of public engagement. Particularly, the evidence for the significant moderations by PBC in a context that is dependent on skills to fulfill a range of goals for the behavior (i.e., goals for communication in public engagements) emphasizes the importance of high levels of PBC as the elevators of behavioral outcomes. Importantly, the results also provide evidence that the moderating effect exists for the relationship between personal norms and scientists’ public engagement. This is an insightful finding on the moderating effect of PBC on a norms-willingness relationship since the construct of perceived social norms has been the more common predictors in the TPB. Earlier on, we highlighted that some empirical studies on the moderating effects of PBC could not find substantial support for the proposition. Based on the significant moderations of not only attitude and personal norms but explications of the perceived social norms for public engagement, this study adds to the evidential base from the recent years of the proposed moderating effects (e.g., [62, 74, 83, 84]).

The significant relationships involving PBC bear important implications for organizers of public engagement training in light of past findings that suggested that training was associated with the perceived ability to conduct public engagement and the willingness to do so [85, 86]. Building on the arguments on the viability of providing training programs in public engagement to raise PBC, the significant moderations suggested that the content of the training programs can be tailored to scientists who hold less favorable attitude toward, who perceived weaker social norms of, or who possess weaker personal norms for public engagement. To boost public engagement among these groups of scientists, organizers of training programs can identify and target the training programs at them. Better provisions of learning opportunities can provide the impetus for public engagement among these scientists as they try to make up for the less favourable assessments of public engagement. When scientists are adequately trained to the extent that they perceive themselves to be able to carry out public engagement with greater ease, there is a greater likelihood that they would be able to fulfill their roles scientists who contribute to the welfare of their society.

Based on the positive association between personal norms and scientists’ public engagement, it is crucial for the scientific community to note that more scientists could be taking on the identity of a civic scientists when they reach out to the public. This likelihood may come as an encouraging phenomenon for the supporters of the notion of civic scientists (e.g., [87, 88]). Research institutes who wish to align their mission with the civic role of science can tap on scientists’ intrinsic motivation for public engagement and support them by providing more avenues for them to fulfill their personal norms. For instance, research institutes can provide more recommendations for scientists to serve their local communities or inform public policies through collaborations with external organizations. To sustain or promote the organizational culture of public engagement as a service to society, research institutes can consider setting up mentoring programs for scientists with strong personal norms for public engagement to nurture others into sharing their understanding on impact of science on humanity. Research institutes can collaborate with trainers of public engagement programs for university students by having such scientists inspire students to do the same. Structural changes at the organizational level need to be implemented to help the scientists with strong moral inclinations for public engagement to translate their strong personal norms into actions.

### Limitations of study and recommendations for future research

There are several limitations to our study. First, with the use of cross-sectional survey data, this study was not able to establish causal relationships. Second, this study utilized self-report data for the analysis. In doing so, social desirability bias [89] could have influenced the respondents to provide favorable answers in this study context so that they would portray themselves better. To reduce this possibility, we administered the questionnaire online and informed the potential respondents of anonymity and confidentiality in the recruitment e-mails. Third, self-selection bias could have affected the representativeness of the data in terms of capturing the responses of scientists who were more interested in the topic. To minimize the influence of self-selection bias, we avoided evaluating public engagement in the recruitment e-mail.

We offer several recommendations for future studies in relation to limitations of the measures. The measure for the criterion variable—willingness to conduct public engagement—was not presented in a manner that was directed at a particular behavior, but a across multiple behavioral items instead. Future studies should measure the criterion variable using a simpler method: provide a single description of the behavioral category. Other variables can then be examined in relation to the target behavior defined [90].

Also pertaining to the criterion variable, references to the context—public engagement of science in Singapore—were not explicit in the measures, as per the principles of compatibility of the TPB [91]. Therefore, future studies should make specific references to the context of the criterion variable, alongside the target, action and time elements. Relatedly, wordings for the predictor constructs should also comply with the principles of compatibility. Meanwhile, the wordings within the predictors that were used to refer to the target behaviors need to be kept consistent across all constructs considered (e.g., PBC: “Within the next 12 months, I am confident that I can *carry out public engagement on a topic related to my research for a group of non-expert public* in Singapore.” and perceived descriptive norms: “Within the next 12 months, most scientists like me would *carry out public engagement on a topic related to their research for a group of non-expert public* in Singapore.”).

It is also possible that future studies adapt measures from the prototype willingness model [92]—which considered the willingness construct—by first establishing a scenario for public engagement (e.g., “Suppose within the next 12 months, you receive a request to perform public engagement on a topic related to your research for a group of non-expert public in Singapore.”) and requiring respondents to rate their level of willingness to perform the public engagement on a Likert scale. Related to the abovementioned limitation posed by the usage of cross-sectional survey data, it is also possible that future studies carry out longitudinal studies by measuring the criterion variable at a time period after the predictors.

Informed by studies that showed that scientists conduct public engagement over a variety of online and offline platforms (e.g., [93, 94]), we suggest that future studies explicate scientists’ public engagement into the dimensions of social media, mass media, and face-to-face communication. This research direction can provide further insights on the moderating effect of PBC for public engagements over various types of communication platforms. Next, one limitation is that our operationalization of PBC in terms of skills did not allow us to provide recommendations for research institutes to promote public engagement as an organizational culture through the provision of time, funding, and organizational assistance. Scholars who wish to further research on the moderating effects of PBC can also examine how dimensions of PBC, for example the availability of time, financial resources, and organization assistance, would moderate the predictors of public engagement. Based on the earlier discussion on personal norms, civic scientists, and engagement objectives, another prospective direction for future research is an examination of how personal norms for public engagement influences scientists’ willingness to conduct various types of public engagement that would fulfill their objectives.

## Conclusion

This study sets out to examine the moderating effect of PBC on the relationships between the TPB variables and scientists’ public engagement. Given the significant moderations, this study has contributed to the literature of scientists’ public engagement by showing that PBC can elevate scientists’ public engagement at their current levels of attitude and norms. This is an important finding for the scholarship on scientists’ public engagement because the relevant stakeholders can design programs for various groups of scientists to raise their perceived self-efficacy to conduct public engagement. With more skillful scientists who are willing to carry out public engagement, the lay public will get more opportunities to engage with science through public engagement.

## Supporting information

S1 Table Measurement items and descriptive statistics. (DOCX)

S2 Table Zero-order correlations of all variables. (DOCX)

## References

[1] Hazelton A. Why is scientific collaboration key? 4 experts explain: World Economic Forum; 2021 [cited 2021 Jun 20]. Available from: https://www.weforum.org/agenda/2021/06/4-views-on-why-scientific-collaboration-is-key-for-the-future/

[2] Dudo A, Besley JC. Scientists’ prioritization of communication objectives for public engagement. PLoS ONE. 2016; 11(2): e0148867. doi: 10.1371/journal.pone.0148867 26913869 PMC4767388

[3] Singh GG, Tam J, Sisk TD, Klain KC, Mach ME, Martone RG, et al. A more social science: Barriers and incentives for scientists engaging in policy. Frontiers in Ecology and the Environment. 2014; 12(3): 161–6.

[4] Rowe G, Frewer LJ. A typology of public engagement mechanisms. Science, Technology, & Human Values. 2015; 30(2): 251–90.

[5] Bauer MW, Jensen P. The mobilization of scientists for public engagement. Public Understanding of Science. 2011; 20(1): 3–11.

[6] Besley JC, Dudo A, Storksdieck M. Scientists’ views about communication training. Journal of Research in Science Teaching. 2015; 52(2): 199–220.

[7] Poliakoff E, Webb TL. What factors predict scientists’ intentions to participate in public engagement of science activities? Science Communication. 2007; 29(2): 242–63.

[8] Rowe G, Marsh R, Frewer LJ. Evalution of a deliberative conference. Science, Technology, & Human Values. 2004; 29(1): 88–121.

[9] Tsfati Y, Cohen J, Gunther AC. The influence of presumed media inflience on news about science and scientists. Science Communication. 2011; 33(2): 143–66.

[10] Besley JC, Oh SH, Nisbet M. Predicting scientists’ participation in public life. Public Understanding of Science. 2012; 22(8): 971–87. doi: 10.1177/0963662512459315 23825262

[11] Bucchi M. Of deficits, deviations and dialogues: Theories of public communication of science. In: Bucchi M, Trench B, editors. Handbook of Communication of Science and Technology. Routledge; 2008. p. 57–76.

[12] Rose KM, Howell EL, Scheufele DA, Brossard D, Xenos MA, Shapira P, et al. The values of synthetic biology: Researcher views of their field and participation in public engagement. BioScience. 2018; 68(10): 782–91.

[13] Chodos A. Scientists issue call for more public engagement. American Physical Society; 2009 [cited 2021 Jul 11]. Available from: https://www.aps.org/publications/apsnews/200906/public.cfm.

[14] Holt RD. Why Science? Why AAAS? Science. 2015; 347(6224): 807. doi: 10.1126/science.aaa9126 25700491

[15] Metcalfe J, Alford K, Shore J. National audit of Australian science engagement activities, 2012. Econnect Communication, Bridge8 and Australian Science Communicators; 2012.

[16] National Academies of Sciences, Engineering, and Medicine. Communicating science effectively: A research agenda; 2017. 28406600

[17] Varner J. Scientific outreach: Toward effective public engagement with biologial science. BioScience. 2014; 64(4): 333–40.

[18] Kaye DK, Bakyawa J, Kakande N, Sewankambo N. The media’s and health scientists’ perceptions of strategies and priorities for nurturing positive scientist-media interaction for communicating health research in Uganda. Journal of Media and Communication Studies. 2011; 3(3): 112–7.

[19] Cerrato S, Daelli V, Pertot H, Puccioni O. The public-engaged scientists: Motivations, enablers and barriers. Research for All. 2018; 2(2): 313–22.

[20] Ecklund EH, James SA, Lincoln AE. How academic biologists and phycists view science outreach. PLoS ONE. 2012; 7: e36240. 22590526 10.1371/journal.pone.0036240 PMC3348938

[21] Powell MC, Colin M. Meaningful citizen engagement in science and technology: What would it really take? Science Communication. 2008; 30(1): 126–36.

[22] Burchell K. Factors affecting public engagement by researchers: Literature review. Policy Studies Institute, London; 2015.

[23] Hamlyn B, Shanahan M, Lewis H, O’Donoghue E, Hanson T, Burchell K. Factors affecting public engagement by researchers: A study on behalf of a Consortium of UK public research funders. TNS-BMRB and the Policy Studies Institute; 2015.

[24] Besley JC, Dudo A, Yuan S, Lawrence F. Understanding scientists’ willingness to engage. Science Communication. 2018; 40(5): 559–90.

[25] Dudo A. Toward a model of scientists’ public communication: The case of biomedical researchers. Science Communication. 2012; 35(4): 476–501.

[26] Besley JC. What do scientists think about the public and does it matter to their online engagement? Science and Public Policy. 2015; 42(2): 201–14.

[27] Dunwoody S, Brossard D, Dudo A. Socialization or rewards? Predicting US scientists-media interactions. Journalism & Mass Communication Quarterly. 2009; 86(2): 299–314.

[28] Dudo A, Besley J, Kahlor LA, Koh H, Copple J, Yuan S. Microbiologists’ public engagement views and behaviors. Journal of Microbiology & Biology Education. 2018; 19(1): 19.1.28. doi: 10.1128/jmbe.v19i1.1402 29904524 PMC5969410

[29] Bandura A. Self-efficacy: The exercise of control. New York: Freeman; 1977.

[30] Ajzen I. The theory of planned behavior. Organizational Behavior and Human Decision Processes. 1991; 50(2): 179–211.

[31] Ajzen I. The theory of planned behavior: Frequently asked questions. Human Behavior & Emerging Technology. 2020; 2(4): 314–24.

[32] Ajzen I. From intentions to actions: A theory of planned behavior. In: Kuhl J, Beckmann J, editors. Action control: From cognition to behaviour. Berlin, Heidelberg: Springer; 1985. p. 11–39.

[33] Sniehotta FF, Presseau J, Araújo-Soares V. Time to retire the theory of planned behavior. Health Psychology Review. 2014; 8(1): 1–7. 25053004 10.1080/17437199.2013.869710

[34] Ajzen I. The theory of planned behaviour: Reactions and reflections. Psychology & Health. 2011; 26(9): 1113–27. doi: 10.1080/08870446.2011.613995 21929476

[35] Kruse P, Wach D, Wegge J. What motivates social entrepreneurs? A meta-analysis on predictors of the intention to found a social enterprise. Journal of Small Business Management. 2021; 59(3): 477–508.

[36] Zaremohzzabieh Z, Ahrari S, Krauss SE, Abu Samah A, Meng LK, Ariffin Z. Predicting social entrepreneurial intention: A meta-analytic path analysis based on the theory of planned behavior. Journal of Business Research. 2019; 96: 264–276.

[37] La Barbera F, Ajzen I. Moderating role of perceived behavior control in the theory of planned behavior: A preregistered study. Journal of Theoretical Social Psychology. 2021; 5(1): 35–45.

[38] Andrews E, Weaver A, Hanley D, Shamatha J, Melton G. Scientists and public outreach: Participation, motivations, and impediments. Journal of Geoscience Education. 2005; 53(3): 281–93.

[39] Gavhi-Molefe MR, Jensen E, Joubert M. Why scientists agree to participate in science festivals: Evidence from South Africa. International Journal of Science Education, Part B: Communication and Public Engagement. 2021; 11(2): 127–42.

[40] Qi L, Liu X, Ren F, editors. Studies on scientists engagement in public outreach in China: Motivations, impediments, and countermeasures. Portland International Conference on Management of Engineering and Technology (PICMET); 2013; San Jose, CA, USA.

[41] Ho SS, Looi J, Goh TJ. Scientists as public communicators: Individual- and institutional-level motivations and barriers for public communication in Singapore. Asian Journal of Communication. 2020; 30(2): 155–78.

[42] Pomery EA, Gibbons FX, Reis-Bergan M, Gerrard M. From willingness to intention: Experience moderates the shift from reactive to reasoned behavior. Personality and Social Psychology Bulletin. 2009; 35(7): 894–908. doi: 10.1177/0146167209335166 19429884 PMC2742327

[43] Gibbons FX, Gerrard M, Blanton H, Russell DW. Reasoned action and social reaction: Willingness and intention as independent predictors of health risk. Journal of Personality and Social Psychology. 1998; 74(5): 1164–80. doi: 10.1037//0022-3514.74.5.1164 9599437

[44] National Research Foundation. Research Innovation Enterprise 2020 Plan: Winning the future through science and technology. Prime Minister Office, Singapore; 2016.

[45] de Souza DE, Zhao LZ, Mani L, Toh G, Lin B. Singapore: An evolving and increasingly complex relationship. In: Gascoigne T, Schiele B, Leach J, Riedlinger M, Lewenstein BV, Massarain L, et al., editors. Communicating science: A global perspective: Australian National University Press; 2020. p. 743–69.

[46] Institute for Health Innovation & Technology. Science communication workshop for public engagement. Singapore: National University of Singapore; 2021. [cited Jun 30]/ Available from: https://ihealthtech.nus.edu.sg/science-communication-workshop-for-public-engagement/.

[47] Longnecker N, Gondwe M. Graduate degree programmes in science communication: Educating and training science communicators to work with communities. In: Hin LTW, Subramaniam R, editors. Communicating Science to the Public. Springer; 2014. p. 141–160.

[48] Ho SS, Looi J, Leung YW, Goh TJ. Public engagement by researchers of different disciplines in Singapore: A qualitative comparison of macro- and meso-level concerns. Public Understanding of Science. 2019; 29(2): 211–29. doi: 10.1177/0963662519888761 31778090 PMC7323768

[49] Cerrato S, Daelli V, Pertot H, Puccioni O. The public-engaged scientists: Motivations, enablers and barriers. Research for All. 2018; 2(2): 313–322.

[50] Buxner SR, Sharma M, Hsu B, Peticolas L, Nova MAM, CoBabe-Ammann E. Barriers, lessons learned, and best practices in engaging scientists in education and public outreach. ASP Conference Series. 2012; 457: 81–87.

[51] Entradas M, Bauer MM. Mobilisation for public engagement: Benchmarking the practices of research institutes. Public Understanding of Science. 2017; 26(7): 771–788. doi: 10.1177/0963662516633834 26951156

[52] Ho SS, Yang X, Liao Y, Turner D, Tan R, Chan JM. A survey of public views and attitude towards science and technology issues in Singapore. Asian Scientist; 2015.

[53] Eagly A, Chaiken S. Attitude strength, attitude structure and resistance to change. In: Petty R, Kosnik J, editors. Attitude Strength. Erlbaum; 1995. p. 413–32.

[54] Agnella S, de Bortoli A, Scamuzzi S. How and why scientists communicate with society: The case of physics in Italy. In: Bucchi M, Trench B, editors. Quality, Honesty and Beauty in Science and Technology Communication: PCST 2012 Book of Papers (Proceedings of the 12th International Conference “Public Communication of Science and Technology”, Florence, Italy, 18–20 April 2012). Vicenza: Observa Science in Society; 2012. p. 377–81.

[55] The Royal Society. Factors affecting science communication: A survey of scientists and engineers. 2006.

[56] Lapinski MK, Rimal RN. An explication of social norms. Communication Theory. 2005; 15(2): 127–47.

[57] Martín-Sempere M, Garzón-García B, Rey-Rocha J. Scientists’ motivation to communicate science and technology to the public: Surveying participants at the Madrid Science Fair. Public Understanding of Science. 2008; 17(3): 349–67.

[58] Dudo A, Kahlor L, AbiGhannam N, Lazard A, Liang M. An analysis of nanoscientists as public communicators. Nature Nanotechnology. 2014; 9: 841–4. doi: 10.1038/nnano.2014.194 25218326

[59] Mizumachi E, Matsuda K, Kano J, Kawakami M, Kato K. Scientists’ attitudes toward a dialogue with the public: A study using “science cafes”. Journal of Science Communication. 2011; 10(4): 1–11.

[60] Parker D, Manstead AS, Stradling SG. Extending the theory of planned behaviour: The role of personal norm. British Journal of Social Psychology. 1995; 34(2): 127–38.

[61] Schwartz SH. Normative explanations of helping behavior: A critique, proposal, and empirical test. Journal of Experimental Social Psychology. 1973; 9(4): 349–64.

[62] Onwezen MC, Antonides G, Bartels J. The norm activation model: An exploration of the functions of anticipated pride and guilt in pro-environmental behaviour. Journal of Economic Psychology. 2013; 39: 141–53.

[63] Bentley P, Syvik S. Academic staff and public communication: A survey of popular science publishing across 13 countries. Public Understanding of Science. 2011; 20(1): 48–63.

[64] Sharman A, Howarth C. Climate stories: Why do climate scientists and sceptical voices participate in the climate debate? Public Understanding of Science. 2017; 26(7): 826–42. doi: 10.1177/0963662516632453 26969714

[65] Madden TJ, Ellen PS, Ajzen I. A comparison of the theory of planned behavior and the theory of reasoned action. Personality and Social Psychology Bulletin. 1992; 18(1): 3–9.

[66] Ajzen I, Driver BL. Application of the theory of planned behavior to leisure choice. Journal of Leisure Research. 1992; 24(3): 207–24.

[67] Yzer M. Does perceived control moderate attitudinal and normative effects on intention? A review of conceptual and methodological issues. In: Ajzen I, Albarracin D, Hornik R, editors. Prediction and change of health behavior: Applying the reasoned action approach. Erlbaum; 2007. p. 107–23.

[68] Yzer M, van den Putte B. Control perceptions moderate attitudinal and normative effects on intention to quit smoking. Psychology of Addictive Behaviors. 2014; 28(4): 1153–61. doi: 10.1037/a0037924 25243830

[69] Fishbein M, Ajzen I. Predicting and changing behavior: The reasoned-action approach. New York: Psychology Press; 2010.

[70] Ajzen I. Perceived behavioral control, self-efficacy, locus of control, and the theory of planned behavior. Journal of Applied Social Psychology. 2002; 32(4): 665–83.

[71] Martinez LS, Lewis N. The moderated influence of perceived behavioral control on intentions among the general US population: Implications for public communication campaigns. Journal of Health Communication. 2016; 21(9):1006–15. 27565188 10.1080/10810730.2016.1204378 PMC5042688

[72] Conner M, McMillan B. Interaction effects in the theory of planned behaviour: Studying cannabis use. British Journal of Social Psychology. 1999; 38(2): 195–222. doi: 10.1348/014466699164121 10392450

[73] Umeh K, Patel R. Theory of planned behaviour and ecstasy use: An analysis of moderator‐interactions. British Journal of Health Psychology. 2004; 9(1): 25–38. doi: 10.1348/135910704322778704 15006199

[74] Castanier C, Deroche T, Woodman T. Theory of planned behaviour and road violations: The moderating influence of perceived behavioural control. Transportation Research Part F: Traffic Psychology and Behaviour. 2013; 18: 148–158.

[75] Kreimer P, Levin L, Jensen P. Popularization by Argentine researchers: The activities and motivations of CONICET scientists. Public Understanding of Science. 2011; 20(1): 37–47.

[76] Schultz PW, Messina A, Tronu G, Limas EF, Gupta R, Estrada M. Personalized normative feedback and the moderating role of personal norms: A field experiment to reduce residential water consumption. Environment and Behavior. 2014; 48(5): 1–25.

[77] Hagger MS, Cheung MW-L, Ajzen I, Hamilton K. Perceived behavioral control moderating effects in the theory of planned behavior: A meta-analysis. Health Psychology. 2022; 41(2): 155–167. doi: 10.1037/hea0001153 35143225

[78] Clark F, Illman DL. Dimensions of civic science. Science Communication. 2001; 23(1): 5–27.

[79] Greenwood MRC, Riordan DG. Civic scientist/civic duty. Science Communication. 2001; 23(1): 28–40.

[80] Deci EL, Ryan RM. Intrinsic motivation and self-determination in human behavior. New York: Plenum; 1985.

[81] Besley JC, Newman TP, Dudo A, Tiffany LA. Exploring scholars’ public engagement goals in Canada and the United States. Public Understanding of Science. 2020; 29(8): 855–67. doi: 10.1177/0963662520950671 32878551

[82] Besley JC, Dudo A, Yuan S. Scientists’ views about communication objective. Public Understanding of Science. 2017; 27(8): 708–30. 28841818 10.1177/0963662517728478

[83] Earle AM, Napper LE, LaBrie JW, Brooks-Russell A., Smith D, de Rutte J. Examining interactions within the theory of planned behavior in the prediction of intentions to engage in cannabis-related driving behaviors. Journal of American College Health. 2019; 68(4): 374–80. doi: 10.1080/07448481.2018.1557197 30681931 PMC6658360

[84] Hukkelberg SS, Hagtvet KA, Kovac VB. Latent interaction effects in the theory of planned behavior applied to quitting smoking. British Journal of Health Psychology. 2014; 19(1): 83–100. 23398564 10.1111/bjhp.12034

[85] Copple J, Bennett N, Dudo A, Moon W-K, Newman TP, Besley J, et al. Contribution of training to scientists’ public engagement intention: A test of indirect relationships using parallel multiple mediation. Science Communication. 2020; 42(4): 508–37.

[86] Stylinski C, Storksdieck M, Canzoneri N, Klein E, Johnson A. Impacts of a comprehensive public engagement training and support program on scientists’ outreach attitudes and practices. International Journal of Science Education, Part B: Communication and Public Engagement. 2018; 8(4): 340–54.

[87] Hendricks R. Scientific societies ad civic science: Current landscape and the future. The Center for Advancement of Information Science Education; 2020 [cited 2021 Jul 10]. Available from: https://www.informalscience.org/news-views/scientific-societies-and-civic-science-current-landscape-and-future.

[88] Cheng Y. The civic duties of scientists. Bulletin of the Atomic Scientists; 2017 [cited 2021 Jul 19]. Available from: https://thebulletin.org/2017/02/the-civic-duties-of-scientists/.

[89] Johnson TP, Fendrich M, Mackesy-Amiti ME. An evaluation of the Crowne-Marlowe social desirability scale. Quality & Quantity. 2012; 46(6): 1883–96.

[90] Ajzen I. University of Massachusetts Amherst; [cited 2022 August 15]. Available from https://people.umass.edu/aizen/faq.html

[91] Ajzen I. Attitudes, personality, and behavior. London: Open University Press; 1988.

[92] Gibbons FX, Gerrard M. Health images and their effects on health behavior. In: Buunk BP, Gibbons FX, editors. Health, coping, and well-being: Perspectives from social comparison theory. Lawrence Erlbaum Associates; 1997. p. 63–94.

[93] Collins K, Shiffman D, Rock J. How are scientists using social media in the workplace? PLoS ONE. 2016; 11(10): e0162680. doi: 10.1371/journal.pone.0162680 27732598 PMC5061391

[94] Mayhew MA, Hall MK. Science communication in a Café Scientifique for high school teens. Science Communication. 2012; 34(4): 546–54.

