# Supplementary material for: Perceived behavioral control as a moderator: Scientists’ attitude, norms, and willingness to engage the public
Source: PLoS One. 2022 Oct 5;17(10):e0275643. doi: 10.1371/journal.pone.0275643 (PMC9534423; doi:10.1371/journal.pone.0275643)
Supplement: S1 Table — (DOCX) [file pone.0275643.s001.docx]

**Supporting Information**

| **Table S1. Measurement Items and Descriptive Statistics** | | | |
| --- | --- | --- | --- |
| Items | *M* | *SD* | Cronbach’s α |
| *Willingness to conduct public engagement* | 3.72 | 0.72 | .92 |
| On a scale of 1 to 5 (1 = not at all willing; 5 = very willing), how willing are you to perform these behaviors in the next 12 months?    In the next 12 months, I am willing to… |  |  |  |
| 1. Be interviewed by media targeted at the non-expert public on science topics. | 3.59 | 1.17 |  |
| 1. Write an article to share science knowledge on a non-scholarly publication (e.g., newspapers, magazines, etc). | 3.73 | 1.10 |  |
| 1. Answer science questions from a journalist (face-to-face, via phone or e-mail). | 3.82 | 0.98 |  |
| 1. Provide science knowledge to the public relations department of research institutions. | 4.07 | 0.91 |  |
| 1. Write an opinion piece on science issues for the newspaper. | 3.49 | 1.12 |  |
| 1. Use my personal social media accounts (e.g., Facebook, Instagram, blogs) to share science knowledge. | 3.05 | 1.39 |  |
| 1. Use my personal social media accounts (e.g., Facebook, Instagram, blogs) to post my own thoughts or comments on science issues. | 2.90 | 1.33 |  |
| 1. Respond to online discussions by the non-expert public regarding my research. | 3.46 | 1.17 |  |
| 1. Answer a query on science from a non-expert member of the public via e-mail. | 3.77 | 1.09 |  |
| 1. Speak to primary and secondary school teachers to share science knowledge. | 3.97 | 1.01 |  |
| 1. Discuss policies related to science issues with policy-makers. | 3.91 | 1.02 |  |
| 1. Deliver science talks at non-governmental organizations. | 3.94 | 0.97 |  |
| 1. Deliver science talks at non-tertiary education institutions (e.g., primary schools and secondary schools). | 3.88 | 1.04 |  |
| 1. Share science knowledge with lay visitors to my institution. | 4.13 | 0.85 |  |
| 1. Bring non-expert members of the public on a lab tour. | 3.87 | 1.01 |  |
|  |  |  |  |
|  |  |  |  |
|  |  |  |  |
|  |  |  |  |
| Items | *M* | *SD* | Cronbach’s α |
| 1. Engage the non-expert public at a public science event (e.g., open house days, science fairs, public exhibitions, public meetings, conferences for the non-expert public). | 3.93 | 0.94 |  |
|  |  |  |  |
| *Attitude toward public engagement* | 4.10 | 0.77 | .90 |
| Communicating my research findings to the non-expert public is... |  |  |  |
| 1. Bad : Good | 4.21 | 0.87 |  |
| 1. Unenjoyable : Enjoyable | 4.05 | 0.89 |  |
| 1. Pointless : Worthwhile | 4.10 | 0.91 |  |
| 1. Unpleasant : Pleasant | 4.05 | 0.85 |  |
|  |  |  |  |
| *Perceived negative external norms* | 3.77 | 0.85 | .74 |
| On scale of 1 to 5 (1 = not important at all; 5 = very important), how important are the following considerations when you correspond with the media or carry out public engagement of science for your field of research? |  |  |  |
| 1. Possible critical reactions from other scientists in the same field. | 3.87 | 1.01 |  |
| 1. Possible critical reactions from my supervisor(s). | 3.93 | 1.08 |  |
| 1. Possible critical reactions from the non-expert public. | 3.50 | 1.03 |  |
|  |  |  |  |
| *Perceived positive media influence* | 3.55 | 0.70 | .79 |
| On a scale of 1 to 5 (1 = strongly disagree; 5 = strongly agree), how far do you agree with the following statements? |  |  |  |
| 1. Universities find it more difficult to deny tenure to candidates who make frequent media appearances. | 3.15 | 0.90 |  |
| 1. Published research that also appears in the mass media (e.g., news, TV programs) gets more attention from other researchers. | 4.00 | 0.85 |  |
| 1. Published research that also appears in the mass media (e.g., news, TV programs) tends to be more frequently cited. | 3.44 | 1.01 |  |
| 1. Media appearances help researchers get funding. | 3.58 | 0.98 |  |
| 1. Media appearances help researchers receive the praise of their colleagues. | 3.56 | 1.03 |  |
|  |  |  |  |
|  |  |  |  |
|  |  |  |  |
|  |  |  |  |
| Items | *M* | *SD* | Cronbach’s α |
| *Perceived descriptive norms* | 3.63 | 0.83 | .84 |
| On scale of 1 to 5 (1 = strongly disagree; 5 = strongly agree), how much do you agree or disagree with the following statements? |  |  |  |
| 1. My colleagues take part in public engagement of science. | 3.53 | 0.97 |  |
| 1. My superiors take part in public engagement of science. | 3.58 | 1.03 |  |
| 1. Other scientists in Singapore take part in public engagement of science. | 3.77 | 0.86 |  |
|  |  |  |  |
| *Personal norms* | 3.52 | 0.87 | .84 |
| On scale of 1 to 5 (1 = strongly disagree; 5 = strongly agree), how much do you agree or disagree with the following statements? |  |  |  |
| 1. I feel a personal obligation to engage in public engagement of science. | 3.57 | 1.04 |  |
| 1. I feel morally obliged to engage in public engagement of science, regardless of what others do. | 3.51 | 1.05 |  |
| 1. I feel guilty when I turn down public engagement requests. | 3.29 | 1.06 |  |
| 1. It is important to take part in public engagement activities because taxpayers’ money funds research. | 3.71 | 1.07 |  |
|  |  |  |  |
| *Perceived behavioral control* | 3.90 | 0.71 | .88 |
| Based on your own skill set, how confident (1 = not confident at all; 5 = very confident) are you in carrying out the following? |  |  |  |
| 1. Explain scientific facts in a way the non-expert public can understand. | 3.99 | 0.83 |  |
| 1. Adapt to different groups of people who are non-expert. | 3.89 | 0.81 |  |
| 1. Anticipate what the non-expert public might want to know about my field of research. | 3.73 | 0.87 |  |
| 1. Answer questions posed to me by the non-expert public. | 4.01 | 0.80 |  |
|  |  |  |  |
