# Supplementary material for: Perceived behavioral control as a moderator: Scientists’ attitude, norms, and willingness to engage the public
Source: PLoS One. 2022 Oct 5;17(10):e0275643. doi: 10.1371/journal.pone.0275643 (PMC9534423; doi:10.1371/journal.pone.0275643)
Supplement: S2 Table — (DOCX) [file pone.0275643.s002.docx]

**Table S2. Zero-Order Correlations of All Variables**

|  | Variables | 1 | 2 | 3 | 4 | 5 | 6 | 7 |
| --- | --- | --- | --- | --- | --- | --- | --- | --- |
| 1 | Attitude | - |  |  |  |  |  |  |
| 2 | Perceived descriptive norms | .26** | - |  |  |  |  |  |
| 3 | Perceived positive media influence | .21** | .24** | - |  |  |  |  |
| 4 | Perceived negative external norms | .20** | .23** | .23** | - |  |  |  |
| 5 | Personal norms | .52** | .34** | .28** | .20** | - |  |  |
| 6 | Perceived behavioral control | .55** | .19** | .20** | .10** | .39** | - |  |
| 7 | Willingness to conduct public engagement | .63** | .22** | .23** | .19** | .55** | .51** | - |

*Note.* **Correlation is significant at the 0.01 level (2-tailed).
